# Terlipressin infusion for prevention of vasoplegic syndrome in patients treated with angiotensin II receptor antagonist undergoing coronary artery bypass graft surgery: a randomized controlled study

**DOI:** 10.1186/s42077-020-0054-6

**Published:** 2020-02-18

**Authors:** Mohamed Saleh, Sherine Kamal Zaki Kodeira, Abdelkhalek Abdelmoneim Aboulseoud

**Affiliations:** Department of Anesthesiology, Intensive Care, and Pain Management, Faculty of Medicine, Ain Shams University, Cairo, Egypt

**Keywords:** Terlipressin, Vasoplegic syndrome, Angiotensin II receptor antagonist, Coronary artery bypass graft surgery

## Abstract

**Background and objectives:**

Preoperative use of renin angiotensin system antagonists has been considered an independent risk factor for development of vasoplegic syndrome. The aim of this study was to demonstrate efficacy of prophylactic terlipressin infusion for prevention of vasoplegic syndrome in patients treated with angiotensin receptor blocker undergoing coronary artery bypass graft surgery.

**Patients and methods:**

One hundred patients on angiotensin II receptor antagonist [losartan] scheduled for coronary artery bypass surgery were enrolled into this prospective randomized controlled study. Anesthetic technique, surgical technique, and cardiopulmonary bypass management were standardized for all patients. With the start of rewarming, patients were randomized to receive either terlipressin infusion 1.3 μg.kg^−1^.hour^−1^, or normal saline infusion. Incidence of vasoplegic syndrome score was used as primary outcome. Hemodynamic parameters, inotropic score, and vasopressor dependency index were used as secondary outcome.

**Results:**

Incidence of vasoplegic syndrome was significantly lower in terlipressin group compared to placebo group. Norepinephrine was required in 2 patients of terlipressin versus 15 patients of placebo group. Mean arterial blood pressure was significantly higher in terlipressin group compared to placebo group (81.7 ± 18.5 versus 69.3 ± 20.2 at 60 min after weaning from CBP). Cardiac index was significantly lower in terlipressin group compared to placebo group (2.52 ± 1.48 versus 3.2 ± 1.55). Systemic vascular resistance was significantly higher in terlipressin group compared to placebo group (2438.09 ± 735.13 versus 1575.05 ± 753.54). Inotropic score and vasopressor dependency index were significantly lower in terlipressin group compared to placebo group.

**Conclusion:**

Prophylactic terlipressin infusion could prevent development of vasoplegic syndrome in patients treated with angiotensin II receptor antagonist undergoing coronary artery bypass graft surgery.

**Trial registration:**

PACTR, PACTR201804003249274, Registered 25/03/2018—retrospectively registered, https://pactr.samrc.ac.za/TrialDisplay.aspx?TrialID=3249.

## Introduction

Vasoplegic syndrome occurring after weaning from cardiopulmonary bypass (CBP) has become an increasingly recognized perioperative issue in cardiac anesthesia. It is characterized by severe hypotension refractory to vasopressor therapy in absence of other identifiable causes (Omar et al., 2015; Fischer & Levin, 2010).

Vasoplegic syndrome could occur in up to 25% of postoperative patients after cardiac surgery. The exact etiology of this syndrome is not clear, and multiple risk factors have been proposed in development of vasoplegic syndrome. However, preoperative use of renin angiotensin system antagonists was considered as an independent risk factor for the development of vasoplegic syndrome (Mekontso-Dessap et al., 2001).

Administration of α adrenergic agonists has been considered the standard management for patients with vasoplegic syndrome occurring after weaning from CBP. However, administration of high doses of α adrenergic agonists has been associated with development of deleterious side effects, including peripheral or mesenteric ischemia (Nygren et al., 2006; Ang et al., 2015).

Vasopressin has been proposed for prophylaxis against development of vasoplegic syndrome (Papadopoulos et al., 2010). However, vasopressin acts on all types of vasopressin receptors. In addition to action on V1 receptors, vasopressin activates V2 receptors on endothelial cells, causing a release of endothelial Von Willebrand factor, which enhances platelet aggregation, and therefore may increase the risk of thrombosis. It also activates V2 receptors in distal renal tubules and collecting ducts, increasing free water reabsorption, and this may lead to development of hyponatremia (Maybauer et al., 2008).

Terlipressin is a long-acting synthetic analog of vasopressin. When compared to vasopressin, terlipressin has a greater selectivity for the V1 receptor and a longer duration of action. Terlipressin has been demonstrated to rapidly correct refractory arterial hypotension after general anesthesia in patients chronically treated with renin angiotensin system antagonists (Boccara et al., 2003). Terlipressin has been studied extensively in patients with septic shock. Several studies demonstrated that continuous infusion of low-dose terlipressin has favorable hemodynamic response (Morelli et al., 2009a; Pesaturo et al., 2006).

The aim of this study was to demonstrate efficacy of prophylactic terlipressin infusion for prevention of vasoplegic syndrome in patients treated with angiotensin receptor blocker undergoing coronary artery bypass graft (CABG) surgery.

## Patients and methods

After obtaining approval from the medical ethics committee, an informed written consent was obtained from all patients before enrollment into this study. One hundred patients scheduled for coronary artery bypass surgery were enrolled into this prospective randomized controlled study.

Inclusion criteria included patients on angiotensin II receptor antagonist losartan for at least 4 weeks before operation, with normal or mild impairment of left ventricular function (EF ≥ 40% by transthoracic echocardiography).

Patients with a critical preoperative state were excluded from the study, including patients with intractable ventricular arrhythmias, previous sudden cardiac arrest, preoperative mechanical ventilation, preoperative use of inotropes, preoperative use of intra-aortic balloon counter-pulsation, and recent myocardial infarction. Patients with previous cardiac surgery, associated valvular heart disease, chronic lung disease, extra-cardiac arteriopathy, and moderate to severe renal impairment were also excluded.

After recording patient’s general characteristics, clinical preoperative data, and preoperative medication, patients were randomly allocated into two equal groups using computer-generated sequence. Allocation concealment was done using sequentially numbered, opaque sealed envelopes. The envelope was opened, and subsequent drug infusion was prepared by the anesthesia consultant in charge. Data collection and data analysis were done by second anesthetist who was blinded to the patient’s allocation group.

All anti-ischemic drugs were continued until the day of surgery including angiotensin II receptor antagonist. Clopidogrel was stopped 5 days before surgery, according to our hospital protocol. Patients followed standard fasting guidelines. Patients were pre-medicated with a 3-mg bromazepam oral tablet the night before surgery, and morphine 0.1 mg.kg^−1^ intramuscular injection 1 h before transferal to the operating room.

On arrival to operative theater, patients were monitored by 5-lead electrocardiogram (lead II, Lead V_5_), and pulse oximetry, using Spacelabs Ultraview SL2700 monitor, Command Module 91496-C (Spacelabs Healthcare, Snoqualmie, WA, USA). Following establishment of peripheral venous access, left radial arterial catheter was inserted after a positive Allen’s test (Benit et al., 1996).

Anesthesia was induced with midazolam 0.1–0.2 mg.kg^−1^, thiopental sodium 3–4 mg.kg^−1^, fentanyl 2–3 μg.kg^−1^, and pancuronium 0.1 mg.kg^−1^. Patients were intubated and connected to mechanical ventilation; Dräger Fabius® OS (Draeger, Inc., Telford, PA, USA). Ventilator was adjusted to deliver tidal volume of 6 mL.kg^−1^, and respiratory rate was adjusted to achieve a partial pressure of carbon dioxide [PaCO2] of 32–36 mmHg.

Following anesthetic induction, 8 French sheath was inserted in right internal jugular vein and a pulmonary artery catheter BD Criticath™ SP5107 (Becton, Dickinson and Company, Franklin Lakes, NJ, USA) was advanced through the sheath. The technique for pulmonary artery catheterization was the same as described by Summerhill and Baram (Summerhill & Baram, 2005). A triple-lumen central venous catheter was inserted in right internal jugular vein beside the sheath as well.

Anesthesia was maintained by isoflurane 0.8–1.2% (expiratory concentration) in 50% oxygen air mixture, and extra analgesia was provided by fentanyl 5 μg.kg^−1^ IV before sternotomy and every hour. Pancuronium in a dose of 0.02 mg.kg^−1^ IV was given every 45 min to maintain adequate muscle relaxation.

Surgical technique and cardiopulmonary bypass management were standardized for all patients. Following median sternotomy and vascular conduit harvesting, systemic anticoagulation was achieved by administration of heparin sulfate 3 mg.kg^−1^ and adjusted to maintain activated clotting time above 480 s. Cardiopulmonary bypass was instituted following aortic and right atrial cannulation, with pump flow rate set at 2.5 L.min^−1^.m^−2^, and mean arterial pressure maintained above 60 mmHg. Antegrade cold crystalloid cardioplegia was administered following cross clamping of the aorta. Tepid hypothermia (32–34° C) was maintained during distal anastomoses. Rewarming was commenced during last distal anastomosis, following which, the aorta was declamped.

With the start of rewarming, patients received the intravenous medication to which they had been randomized. They received either terlipressin (Terlipressin acetate, Glypressin; Ferring Pharmaceuticals, Australia) infusion 1.3 μg.kg^−1^.h^−1^ or equivalent volume of normal saline infusion (0.6 mL.kg^−1^.h^−1^).

Weaning from cardiopulmonary bypass was achieved through optimization of preload, afterload, heart rate, rhythm, and contractility.

In all patients, and both groups:

If mean arterial pressure < 70 mmHg, cardiac index is > 2.5 L.min^−1^.m^−2^, systemic vascular resistance index < 1600 dynes.s.cm^−5^.m^−2^, norepinephrine was infused at a starting dose of 0.03 μg.kg^−1^.min^−1^.

If mean arterial pressure is < 70 mmHg, cardiac index is < 2.5 L.min^−1^.m^−2^, systemic vascular resistance index is > 1600 dynes.s.cm^−5^.m^−2^, epinephrine was infused at a starting dose of 0.03 μg.kg^−1^.min^−1^

The infusion rate was increased by 0.02 μg.kg^−1^.min^−1^ increments every 3 min till stabilization of hemodynamics.

Parameters of the study are as follows:

Incidence of vasoplegic syndrome was used as primary outcome. Vasoplegic syndrome was defined as mean arterial pressure ≤ 70 mmHg, systemic vascular resistance index ≤ 1600 dynes.s.cm^5^.m^−2^, cardiac index ≥ 2.5 L.min^−1^.m^−2^, and central venous pressure ≥ 10 mmHg, immediately after weaning from cardiopulmonary bypass (Mekontso-Dessap et al., 2001).

Hemodynamic parameters, inotropic score, vasopressor dependency index, and clinical outcome were used as secondary outcome. The following hemodynamic parameters were monitored: mean arterial blood pressure, and central venous pressure. Cardiac output (CO) was measured by thermo-dilution technique. The following derived hemodynamic parameters were calculated automatically: cardiac index and systemic vascular resistance index.

$$ \mathrm{Cardiac}\ \mathrm{index}\ \left(\mathrm{CI}\right)=\frac{CO}{BSA\ }\ \mathrm{L}.\min {-}^1.\mathrm{m}{-}^2 $$

$$ \mathrm{The}\ \mathrm{systemic}\ \mathrm{vascular}\ \mathrm{resistance}\ \mathrm{index}\ \left(\mathrm{SVRI}\right)=80\ast \kern0.5em \frac{MAP- CVP}{CI}\ \mathrm{dyn}.\mathrm{s}.\mathrm{cm}{-}^5.\mathrm{m}{-}^2 $$

Hemodynamic parameters were recorded as baseline reading before skin incision, immediately after weaning from CBP, then every 10 min, till 60 min after weaning from CBP.

The inotrope score previously described by Matsukuma and colleagues was adopted to describe the level of inotropic support (Matsukuma et al., 2015).

$$ \mathrm{Inotrope}\ \mathrm{score}=\left(\mathrm{epinephrine}\ \mathrm{dose}\times 100\right)+\left(\mathrm{norepinephrine}\ \mathrm{dose}\times 100\right) $$

All dosages were expressed in micrograms per kilogram per minute.

Vasopressor dependency index (Cruz et al., 2009) was calculated during weaning from CBP according to the following formula:

$$ \mathrm{Vasopressor}\ \mathrm{dependency}\ \mathrm{index}=\kern0.5em \frac{\mathrm{Inotropic}\ \mathrm{score}\ }{MAP} $$

Patients were admitted to cardiac surgery intensive care unit. Group allocation was revealed to ICU staff. Terlipressin infusion was gradually weaned in the ICU according to clinical state of the patient, over a period of 8 h. Routine postoperative management was given to all patients. Decision regarding ventilation and inotrope were based on unit protocol, hemodynamic status and clinical judgment.

The following clinical outcome parameters were also recorded: ICU stay, hospital stay, development of acute kidney injury, major adverse cardiovascular events, and mortality. Acute kidney injury was defined as at least twofold increase in serum creatinine level, or decrease in glomerular filtration rate (GFR) >50%, or decreased urine output less than 0.5 mL.kg^−1^.h^−1^ for 12 h. Major adverse cardiovascular events included non-fatal MI, unstable angina pectoris, heart failure, significant arrhythmia, stroke, new cardiac surgery, or percutaneous coronary intervention.

### Statistical analysis

All data were prospectively collected, coded, and tabulated then subjected to statistical analysis using IBM SPSS Statistics for Windows, Version 20.0 (IBM Corp., Armonk, NY, USA). Normally distributed numerical data were presented as mean ± SD, and differences between groups were compared using the independent Student’s *t* test, data not normally distributed were compared using Mann-Whitney test and are presented as median (IQR) and categorical variables were analyzed using the *χ*^2^ test or fisher exact test and were presented as number (%). All *P* values were two-sided. *P* < 0.05 was considered statistically significant.

Sample size was calculated using PASS 11.0 sample size calculation program; group sample sizes of 46 in group one and 46 in group two achieve 80% power to detect a difference between the group proportions of − 0.2200. The proportion in group one (the treatment group) is assumed to be 0.3000 under the null hypothesis and 0.0800 under the alternative hypothesis. The proportion in group two (the control group) is 0.3000. The test statistic used is the two-sided Z test with pooled variance. The significance level of the test was targeted. Fifty patients were included in each group for possible dropouts.

## Results

**Fig. 1:**
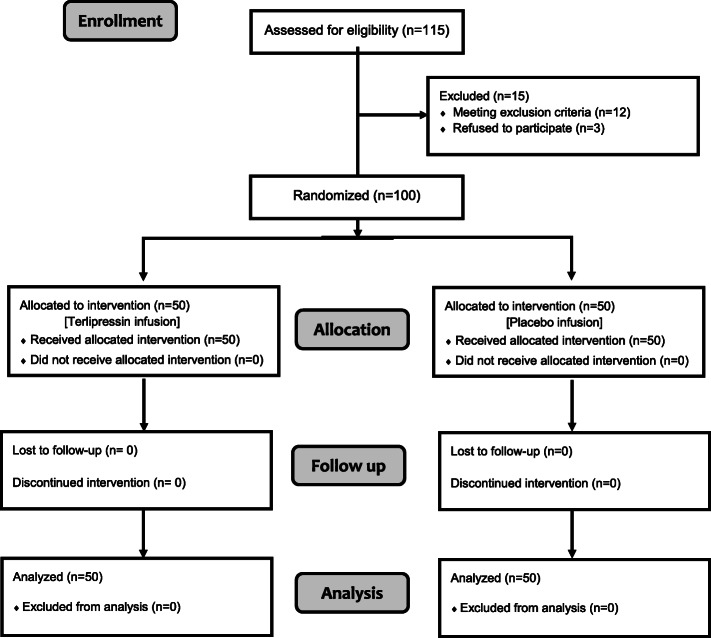
CONSORT flow diagram showing the flow of patients’ participation through each stage of the study

One hundred and fifteen patients were assessed for eligibility for this study. Twelve patients were excluded due to exclusion criteria and three patients refused to participate in the study. Flow of patients’ participation through each stage of this randomized clinical trial was reported according to the consolidated standards of reporting trials (CONSORT) statement (Schulz & Altman, 2010) (Fig. 1).

Patients’ general characteristics, clinical preoperative data, and preoperative medications are shown in Table 1. There was no statistically significant difference between the two groups as regards general characteristics, clinical preoperative data, and preoperative medication.

**Table 1:** General characteristics, clinical preoperative data, and preoperative medication

	Terlipressin group (*n* = 50)	Placebo group (*n* = 50)	*P* value
Age (years)	55.8 ± 6.0	54.4 ± 5.6	0.26
Gender (M/F) (n)	40/10	43/7	0.44
Weight (kg)	71.8 ± 7.3	74.0 ± 8.6	0.17
Height (cm)	160.5 ± 5.7	158.6 ± 6.3	0.10
Ejection Fraction	52.8 ± 8.0	50.6 ± 6.9	0.14
Euro score II	1.1 ± 0.4	1.2 ± 0.3	0.36
Hypertension (*n*)	33	28	0.32
Diabetes mellitus (*n*)	21	26	0.42
Left main disease (*n*)	10	13	0.64
No. of diseased coronaries (2/3)	20/30	17/33	0.54
Losartan dose (mg/d)	52 ± 26.6	53.5 ± 32.3	0.80
β-blocker (*n*)	43	46	0.36
Calcium channel blocker (*n*)	7	5	0.76
Nitrate (*n*)	40	42	0.61
Diuretic (*n*)	9	11	0.63

*n* number of patients, *M/F* male to female ratio

Mean ± SD

*P* value is significant when *P* < 0.05

Intraoperative variables including cardiopulmonary bypass time, aortic cross-clamp time, number of grafts, and lowest nasopharyngeal temperature are shown in Table 2. There was no statistically significant difference between the two groups as regards intraoperative variables.

**Table 2:** Intraoperative variables

	Terlipressin group (*n* = 50)	Placebo group (*n* = 50)	*P* value
CPB time (min)	109.0 ± 34.1	115.1 ± 37.4	0.39
AoX time (min)	38.1 ± 9.6	41.0 ± 8.4	0.10
No. of grafts	3.2 ± 0.8	3.4 ± 0.8	0.32
Hypothermia (° C)	32.8 ± 0.8	33.1 ± 0.9	0.17

*n* number of patients, *CPB* cardiopulmonary bypass, *AoX* aortic cross clamping

Mean ± SD

*P* value is significant when *P* < 0.05

Mean duration of terlipressin infusion was 6.5 ± 1.4 h. The incidence of vasoplegic syndrome was significantly lower in terlipressin group compared to placebo group. Norepinephrine was required in 2 patients of terlipressin versus 15 patients of placebo group. Epinephrine was required in 19 patients of terlipressin versus 21 patients of placebo group. Inotrope score and vasopressor dependency index were significantly lower in terlipressin group than placebo group (Table 3).

**Table 3:** Postoperative variables

		Terlipressin group (*n* = 50)	Placebo group (*n* = 50)	*P* value
NE	Pt (*n*)	2	15	< 0.001*
	Dose (μg.kg^−1^.min^−1^)	0 ± 0.009	0.03 ± 0.042	< 0.001*
E	Pt (*n*)	19	21	0.683
	Dose (μg.kg^−1^.min^−1^)	0.04 ± 0.05	0.05 ± 0.051	0.723
Inotropic score		3 (0–9)	7 (3–11)	0.014*
Vasopressor dependency index		0.06 ± 0.065	0.1 ± 0.074	0.012*

*n* number of patients, *NE* norepinephrine, *E* epinephrine

Mean ± SD

*P* value is significant when *P* < 0.05

As regards mean blood pressure, baseline values were comparable in both terlipressin and placebo group. Following weaning from CBP, mean blood pressure was significantly higher in terlipressin group than placebo group at the different studied time intervals (Table 4).

**Table 4:** Mean arterial blood pressure (mmHg)

	Terlipressin group (*n* = 50)	Placebo group (*n* = 50)	*P* value
Baseline	82.3 ± 10.4	79.7 ± 9.8	0.2013
0 min	71.5 ± 25.3	55.8 ± 24.1	0.0020*
10 min	75.4 ± 20.1	65.3 ± 21.3	0.0165*
20 min	79.2 ± 18.8	69.1 ± 19.5	0.0097*
30 min	80.5 ± 20.4	70.4 ± 20.5	0.0153*
40 min	81.4 ± 19.7	71.3 ± 18.7	0.0099*
50 min	82.1 ± 20.3	70.6 ± 19.3	0.0046*
60 min	81.7 ± 18.5	69.3 ± 20.2	0.0018*

Baseline reading before skin incision

Minutes after weaning from cardiopulmonary bypass

Mean ± SD

*P value is significant when *P* < 0.05

As regards central venous pressure, baseline values were comparable in both terlipressin and placebo group. There was no significant difference between terlipressin group and placebo group at the different studied time intervals after weaning from cardiopulmonary bypass (Table 5).

**Table 5:** Central venous pressure (mmHg)

	Terlipressin group (*n* = 50)	Placebo group (*n* = 50)	*P* value
Baseline	4.2 ± 3.9	4.3 ± 3.7	0.8956
0 min	7.1 ± 6.8	8.6 ± 7.9	0.3114
10 min	6.8 ± 6.7	8.9 ± 8.1	0.1609
20 min	6.9 ± 6.6	8.4 ± 7.9	0.3054
30 min	7.2 ± 6.9	8.7 ± 7.8	0.3109
40 min	6.9 ± 6.7	8.3 ± 7.7	0.3345
50 min	7.1 ± 6.6	8.4 ± 7.4	0.3562
60 min	6.9 ± 6.9	8.3 ± 7.6	0.3372

Baseline reading before skin incision

Minutes after weaning from cardiopulmonary bypass

Mean ± SD

*P value is significant when *P* < 0.05

As regards cardiac index, baseline values were comparable in both terlipressin and placebo group. Following weaning from CBP, cardiac index index was significantly higher in placebo group than terlipressin group at the different studied time intervals (Table 6).

**Table 6:** Cardiac Index (L min^−1^)

	Terlipressin group (*n* = 50)	Placebo group (*n* = 50)	*P* value
Baseline	2.49 ± 0.82	2.67 ± 0.85	0.2838
0 min	2.39 ± 1.53	3.47 ± 1.51	0.0006*
10 min	2.43 ± 1.48	3.25 ± 1.43	0.0059*
20 min	2.47 ± 1.56	3.24 ± 1.49	0.0132*
30 min	2.48 ± 1.47	3.13 ± 1.52	0.0321*
40 min	2.51 ± 1.51	3.19 ± 1.57	0.0296*
50 min	2.56 ± 1.53	3.16 ± 1.49	0.0498*
60 min	2.52 ± 1.48	3.2 ± 1.55	0.0271*

Baseline reading before skin incision

Minutes after weaning from cardiopulmonary bypass

Mean ± SD

*P value is significant when *P* < 0.05

As regards systemic vascular resistance index, baseline values were comparable in both terlipressin and placebo group. Following weaning from CBP, systemic vascular resistance index was significantly higher in terlipressin group than placebo group at the different studied time intervals (Table 7).

**Table 7:** Systemic vascular resistance index (dyn.s.cm^−5^)

	Terlipressin group (*n* = 50)	Placebo group (*n* = 50)	*P* value
Baseline	2573.49 ± 829.26	2319.10 ± 762.35	0.1135
0 min	2222.59 ± 1071.89	1134.29 ± 964.23	< 0.0001*
10 min	2324.29 ± 832.43	1437.53 ± 850.34	< 0.0001*
20 min	2406.47 ± 728.20	1548.14 ± 730.20	< 0.0001*
30 min	2429.03 ± 843.53	1628.11 ± 773.68	< 0.0001*
40 min	2438.24 ± 794.70	1630.09 ± 662.42	< 0.0001*
50 min	2406.25 ± 820.91	1625.31 ± 746.30	< 0.0001*
60 min	2438.09 ± 735.13	1575.05 ± 753.54	< 0.0001*

Baseline reading before skin incision

Minutes after weaning from cardiopulmonary bypass

Mean ± SD

*P value is significant when *P* < 0.05

Despite a significantly lower cardiac index in the terlipressin group, this was not associated with any statistically significant increase in adverse outcomes like prolonged ICU or hospital stay, development of acute kidney injury, major adverse cardiovascular events, or mortality (Table 8).

**Table 8:** Clinical outcome variables

	Terlipressin group (*n* = 50)	Placebo group (*n* = 50)	*P* value
ICU stay (hours)	50.4 ± 10.2	52.7 ± 11.5	0.2927
Hospital Stay (days)	9.8 ± 1.3	10.3 ± 1.5	0.0780
AKI (*n*)	2	4	0.6777
Major adverse CV events (*n*)	3	5	0.7150
Mortality (*n*)	0	1	1

*n* number of patients, *AKI* acute kidney injury defined as at least two fold increase in serum creatinine level, or decrease in GFR > 50%, or decreased urine output less than 0.5 mL.kg^−1^.h^−1^ for 12 h

Mean ± SD

Major adverse cardiovascular events included non-fatal MI, unstable angina pectoris, heart failure, significant arrhythmia, stroke, new cardiac surgery, or percutaneous coronary intervention

*P* value is significant when *P* < 0.05

## Discussion

This randomized controlled study demonstrated that prophylactic terlipressin infusion could prevent development of vasoplegic syndrome in patients treated with angiotensin II receptor antagonist undergoing coronary artery bypass graft surgery. Patient in terlipressin group achieved higher mean arterial pressure, higher systemic vascular resistance index, lower cardiac index, lower inotropic score, and vascular dependency index when compared to placebo group.

In fact, incidence of vasoplegic syndrome following CABG with CBP is variable in literature. While Fischer and Levin reported an incidence ranging between 5 and 25% (Fischer & Levin, 2010), Omar and colleagues reported and a higher incidence ranging from 9 to 44% (Omar et al., 2015).

Renin angiotensin aldosterone system antagonists are widely prescribed for patients with coronary artery heart disease because of their cardioprotective, vasculoprotective, and antiatherogenic properties (Vijayaraghavan & Deedwania, 2011; Ma et al., 2010; Nantel & Rene de Cotret, 2010). However, preoperative use of renin angiotensin aldosterone system antagonist was identified as an important risk factor for development of post-cardiopulmonary bypass vasoplegic syndrome (Mekontso-Dessap et al., 2001). Moreover, the severity of hypotension has been demonstrated to be more pronounced with use of ARBs than angiotensin-converting enzyme (ACE) inhibitors (Yusuf et al., 2008; Oh et al., 2006).

Vasopressin deficiency has been postulated to be one of the mechanisms of post-cardiopulmonary bypass vasoplegic syndrome, thus rendering patients receiving renin angiotensin system antagonists at risk for developing this syndrome (Argenziano et al., 1998; Colson et al., 2011). Vasopressin acts on three types of vasopressin receptors. V1 receptors are present in vascular smooth muscle and mediate vasoconstriction. V2 receptors are present in renal distal tubal and collecting and responsible for the antidiuretic effects of vasopressin. V3 receptors are present in the anterior pituitary and stimulate secretion of adrenocorticotropic hormone (ACTH) (Birnbaumer, 2000).

Use of vasopressin in cardiac surgery has been extensively studied. In 2003, Morales et al. demonstrated in a double-blind randomized controlled study, of 33 patients on angiotensin converting enzyme (ACE) inhibitors scheduled for either CABG or valvular surgery, that prophylactic vasopressin infusion at dose of 0.03 U/min started 20 min before initiation of cardiopulmonary bypass and maintained for a maximum of 3 days or until the patient’s hemodynamics was stable reduced post-cardiopulmonary bypass hypotension and catecholamine requirements and was associated with a shorter intensive care stay (Morales et al., 2003).

In 2010, a similar study performed by Papadopoulos et al. demonstrated that prophylactic administration of vasopressin infusion at a similar dose reduced the incidence of post-CBP vasoplegic syndrome even for patients with low ejection fraction (EF 30–40%) (Papadopoulos et al., 2010).

An interesting study was performed by Hasija et al., who explored cause and effect relationship between ACE inhibitors and vasoplegic shock. They randomized 47 patients scheduled for CABG surgery into three groups: patients who stopped ACE inhibitors 24 h before operation, patients who continued ACE inhibitors until the morning of operation, and patients who continued ACE inhibitors until the morning of operation and received vasopressin infusion from the onset of rewarming. Preoperative ACE inhibitor continuation predisposed to hypotension upon the induction of anesthesia and in the post cardiopulmonary bypass period. Prophylactic low-dose vasopressin infusion prevented post-CBP hypotension (Hasija et al., 2010).

Vasopressin activates V2 receptors on endothelial cells, causing a release of endothelial Von Willebrand factor, which enhances platelet aggregation, and therefore may increase the risk of thrombosis. It also activates V2 receptors in distal renal tubules and collecting ducts, increasing free water reabsorption, and this may lead to development of hyponatremia (Maybauer et al., 2008; Birnbaumer, 2000)

Terlipressin is characterized by greater selectivity for the V1 receptor than vasopressin. The vasopressor (V1 receptor-mediated) to antidiuretic (V2 receptor-mediated) ratios of vasopressin and terlipressin are 1 and 2.2, respectively.

In TERLIVAP study, 45 septic shock patients were randomized to receive either continuous infusion of terlipressin (1.3 μg.kg^−1^.h^−1^), vasopressin (0.03 U.min^−1^), or norepinephrine (15 μg.min^−1^). An additional norepinephrine infusion was given when indicated to maintain MAP at 70 ± 5 mmHg, if this goal was not maintained utilizing the study infusion protocol. The study revealed that terlipressin infusion allowed marked reduction in additional norepinephrine requirement. Moreover, there was no significant difference among the three studied groups in terms of systemic and regional hemodynamic parameters (Morelli et al., 2009b).

In cardiac surgery, in 2009, Noto and colleagues described a series of six patients with vasoplegic retrospective study, and postoperative refractory low SVR hypotension was treated by terlipressin administration in bolus. Exogenous administration of TP normalized SVR and increased the systemic arterial pressure with a minimum effect on pulmonary pressure. Subsequently, the effect on systemic blood pressure enhanced urine output. No major collateral effects were observed (Noto et al., 2009).

In another retrospective study, terlipressin given continuously significantly increased the mean arterial pressure and reduced the heart rate in both groups. Norepinephrine requirements decreased significantly among survivors only. The mean pulmonary artery pressure and pulmonary capillary wedge pressure levels remained unchanged or increased insignificantly (Kunstyr et al., 2008).

In fact, there might be major concern about the safety of terlipressin use on renal, coronary, and tissue perfusion. Terlipressin appears to preserve renal function through maintenance of renal perfusion. In an experimental model of hemorrhagic shock, terlipressin administration was associated with maintained glomerular filtration and tubular function (Cardoso de Castro et al., 2016). Terlipressin administration was associated with improved renal function and induced natriuresis in patients with liver cirrhosis with or without hepatorenal syndrome (Krag et al., 2007; Fabrizi et al., 2009). Terlipressin administration was also associated with improvement of tissue blood flow, with increased blood oxygen saturation and decreased serum lactate level in patients with sepsis (Xiao et al., 2016). Terlipressin administration maintained myocardial perfusion and was not associated with electrocardiography (ECG) changes or regional wall motion abnormality in patients with advanced liver cirrhosis (Krag et al., 2010).

In our study, we have used a low-dose terlipressin infusion; that has been previously studied in patients with septic shock, with favorable hemodynamic response (Morelli et al., 2009b; Xiao et al., 2016), and limited the duration of terlipressin infusion in the ICU.

To our knowledge, this is the first prospective randomized controlled trial demonstrating the hemodynamic effects of terlipressin infusion in patients treated with losartan undergoing CABG surgery.

## Limitation of the study

The current study was limited to intraoperative period, as group allocation was revealed to ICU staff. Authors were concerned that extended uninformed use of terlipressin in postoperative period may be potentially dangerous. Additional studies are needed to determine safety of prolonged terlipressin infusion in cardiac intensive care units. Moreover, due to relatively small sample size, this study might be underpowered to detect adverse outcomes including acute kidney injury, major adverse cardiovascular events, and mortality.

## Conclusion

Prophylactic terlipressin infusion could prevent development of vasoplegic syndrome in patients treated with angiotensin II receptor antagonist undergoing coronary artery bypass graft surgery.

## Data Availability

Individual participant data would be shared after deidentification. Beginning 1 month and ending 12 months following article publication. Data would be available for investigators who provide a methodologically sound proposal. Proposals should be directed to corresponding author.

## Abbreviations

ACE: Angiotensin-converting enzyme
CABG: Coronary artery bypass graft
CBP: Cardiopulmonary bypass
CI: Cardiac index
CO: Cardiac output
ECG: Electrocardiography
EF: Ejection fraction
GFR: Glomerular filtration rate
ICU: Intensive care unit
IQR: Interquartile range
MAP: Mean arterial blood pressure
SD: Standard deviation
SPSS: Statistical Package for Social Science
SVRI: Systemic vascular resistance index
